# Exploring the Effects of *Artemisia absinthium* L. Essential Oil on Pseudopregnancy Model in Rats

**DOI:** 10.1002/vms3.70582

**Published:** 2025-08-22

**Authors:** Mürşide Ayşe Demirel, İpek Süntar, Ali Osman Çeribaşı, Kevser Taban, Gökhan Zengin

**Affiliations:** ^1^ Laboratory Animals Breeding and Experimental Researches Center, Department of Basic Pharmaceutical Sciences, Faculty of Pharmacy Gazi University Ankara Turkey; ^2^ Department of Pharmacognosy, Faculty of Pharmacy Gazi University Ankara Turkey; ^3^ Department of Pathology, Faculty of Veterinary Medicine Fırat University Elazig Turkey; ^4^ Department of Pharmacognosy, Faculty of Pharmacy Sivas Cumhuriyet University Sivas Turkey; ^5^ Department of Biology, Science Faculty Selcuk University Konya Turkey

**Keywords:** *Artemisia absinthium*, Asteraceae, essential oil, in vivo, pseudopregnancy

## Abstract

Pseudopregnancy is a luteal phase syndrome characterized by symptoms of late pregnancy and/or early postpartum periods. This phenomenon is observed in various species, including rats, rabbits and dogs, and arises due to hormonal alterations. Due to the significant side effects of current treatment regimens, there is a growing need for the development of alternative therapeutic strategies. In this context, various plants and natural compounds have been investigated for their potential to modulate hormonal balance, offering a promising avenue for the treatment of pseudopregnancy. *Artemisia absinthium* L., a medicinal plant traditionally utilized as an emmenagogue and abortifacient, is known for its regulatory effect on the dopaminergic system. The study aimed to assess whether *A. absinthium* treatment could restore oestrous cyclicity in pseudopregnant rats. Pseudopregnancy was induced through the administration of a pregnant mare's serum gonadotropin and human chorionic gonadotropin in female rats. The essential oil of *A. absinthium* was administered orally at doses of 12.5, 25 and 50 mg/kg once daily for 10 days, with bromocriptine as a reference treatment. The results indicated that the 25 mg/kg dose of *A. absinthium* essential oil exhibited beneficial effects in the pseudopregnancy model. Gas chromatography analysis revealed that the major components of the essential oil included *cis*‐chrysanthenyl acetate (17.8%), sabinyl acetate (11.6%), terpinen‐4‐ol (6.2%), caryophyllene oxide (5.5%) and (*E*)‐nuciferol (5.5%). These findings suggest that *A. absinthium* essential oil may present a promising therapeutic option for the management of pseudopregnancy.

## Graphical abstract

This study investigated the therapeutic effects of *Artemisia absinthium* essential oil in a rat model of pseudopregnancy. Oral administration of the essential oil, particularly at 25 mg/kg, significantly alleviated pseudopregnancy symptoms, demonstrating comparable efficacy to bromocriptine. GC analysis identified *cis*‐chrysanthenyl acetate and sabinyl acetate as major constituents, suggesting that *A. absinthium* may offer a promising natural alternative for pseudopregnancy management.

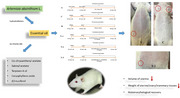

## 1 Introduction

Pseudopregnancy is a luteal phase syndrome that shows clinical signs typical of late pregnancy and/or early postpartum periods despite the absence of pregnancy. This syndrome is characterized by physiological and behavioural effects. Clinically, enlarged mammary glands and lactation can be observed due to increased prolactin levels. In addition to these findings, maternal behaviour occurs during this period (da Silva et al. 2021; Gobello 2021; Falceto et al. 2024). Prolactin levels have been reported to remain elevated for up to 14 days during pseudopregnancy in rats (Freeman et al. 2000; Bertram et al. 2006; Goldman et al. 2007; Kennett and McKee 2012; Cora et al. 2015). However, the potential effects of increased prolactin on maternal behaviour in pseudopregnant rats have not yet been investigated. Diagnosis of pseudopregnancy syndrome is verified by clinical and ultrasonographic examinations. This syndrome is confirmed by the absence of the foetus in the ultrasonographic examination. In addition to these findings, the increase in serum prolactin levels also supports the diagnosis of pseudopregnancy (Tsutsui et al. 2007; Gary and England 2019). If pseudopregnancy is not managed, it may result in mastitis, mammary dermatitis or the development of mammary tumours. As a consequence of this, the cost and duration of the treatment of these cases may increase (Gobello 2021; Cuellar et al. 2024).

The treatment procedures, such as prolactin inhibitors, including dopamine agonists (bromocriptine and cabergoline), serotonin antagonists (metergoline) and progestins, are used to eliminate the behavioural and clinical symptoms (Gobello 2001a; Root et al. 2018; Papich 2021). Although progestins were once widely used for the treatment of pseudopregnancy in dogs, their effectiveness is limited, as pseudopregnancy often recurs after treatment ends. Moreover, the use of progestins can lead to endometrial hyperplasia and may increase the risk of pyometra (Root et al. 2018; Gobello 2021). Bromocriptine (vomiting, anorexia and depression) and metergoline (anxiety, aggressiveness and hyperexcitation) can cause serious side effects (Singh et al. 2018; Gobello 2021). Additionally, these drugs have a short half‐life (Gobello 2021). Although cabergoline is safer than other agents, it is expensive (Root et al. 2018; Gobello 2021). Due to the adverse effects of the current medical treatment regimens in pseudopregnancy, new medical strategies should be developed. Recent studies have suggested that vitamin B6 (pyridoxine) may modulate prolactin secretion, and its use has been investigated for the management of pseudopregnancy‐related symptoms (da Silva et al. 2021; Santos et al. 2024). Natural resources and medicinal plants are of great importance in treating human and animal diseases. In the literature, *Pulsatilla* Mill. (Ranunculaceae), *Ferula* L. (Apiaceae), *Urtica* L. (Urticaceae), *Thuja* L. (Cupressaceae) species and *Matricaria chamomilla* L. (Asteraceae) were reported to be effective for the treatment of pseudopregnancy (Aslan et al. 2004; Özyurtlu and Alaçam 2005; Madrewar and Glencross 2014).

*Artemisia absinthium* L. (wormwood) is an important aromatic and medicinal plant that belongs to the Asteraceae family, used as an emmenagogue and to induce abortus in traditional medicine (Baytop 1999; Judžentienė 2016). The essential oil of *A. absinthium* has high concentrations of terpenes and a wide range of biological activities. It is used as an antimicrobial, antiseptic, anthelmintic, anti‐inflammatory, antidepressant, anti‐cold, carminative, choleretic, digestive and stimulant (Watson and Preedy; Goud and Swamy 2015; Nguyen and Németh 2016). Several studies have shown that *A. absinthium* has a regulatory effect on the dopaminergic system (Sansar and Gamrani 2013; Zeraati et al. 2014; Basiri et al. 2017; Rashidi et al. 2021; El‐Bakry et al. 2024). In our previous study, we investigated the therapeutic effects of petroleum ether, dichloromethane and methanol extracts of *A. absinthium* on the pseudopregnancy model. Among the extracts tested, the petroleum ether extract demonstrated a positive effect, indicating that nonpolar compounds may have a beneficial impact on pseudopregnancy (Demirel et al. 2018). Consequently, the present study seeks to assess the potential activity of *A. absinthium* essential oil, which is rich in nonpolar and volatile compounds. The present study aimed not only to evaluate the endocrine and morphological alterations induced by pseudopregnancy but also to investigate whether *A. absinthium* treatment could promote the resumption of regular oestrous cyclicity in affected animals. This is also the first article to describe the effects of *A. absinthium* essential oil in the treatment of pseudopregnancy in rats.

## 2 Materials and Methods

### 2.1 Plant Materials

The aerial parts of *A. absinthium* L. were collected from Konya (located between Taşkent and Ermenek, Türkiye; GPS coordinates: 36°48′43″ N, 32°50′49″ E, elevation: 1392 m) on 7 July 2019. The plant was identified by Prof. Dr. Evren Yıldıztugay (Faculty of Science, Selçuk University, Konya, Türkiye), and a voucher specimen was deposited in the Department of Biology, Selçuk University (GZ‐1928). The plant material was shade‐dried for 10 days at room temperature and then ground using a laboratory mill. The powdered plant samples were subsequently stored in a dark environment at room temperature.

### 2.2 Essential Oil Analysis

The essential oil was obtained using the hydro‐distillation method. A total of 100 g of dried plant material was distilled in a Clevenger‐type apparatus for 5 h. The essential oil was then dried over anhydrous sodium sulphate, and the resulting oil was stored in an amber vial at +4°C until further analysis.

The composition of the essential oil was characterized using gas chromatography‐flame ionization detector (GC‐FID) and GC–mass spectrometry (GC–MS). GC–MS analysis was conducted with an Agilent 5975 GC–MS system coupled to an Agilent 7890A GC. The chemical components were separated on an HP‐Innowax column (60 m × 0.25 mm, 0.25 µm film thickness). Additional analytical parameters were previously reported in our earlier publication (Ak et al. 2021).

The retention index (RI) was calculated through co‐injection with a homologous series of *n*‐alkanes (C8–C30) under identical experimental conditions. The identification of components was made based on the RI values, as well as the comparison of mass spectra with those in the literature and databases, such as NIST 05 and Wiley 8th Edition.

### 2.3 Animals

Thirty‐six healthy, 29‐day‐old female Sprague–Dawley rats (weighing 70–75 g) were obtained from the Experimental Animal Center at Gazi University (Ankara, Türkiye). Pseudopregnancy can be experimentally induced in immature rats through hormonal administration, as their ovaries predominantly contain antral follicles and lack corpora lutea (CLs). The combined use of pregnant serum gonadotropin (PMSG) and hCG allows for controlled luteal development and has been widely used for this purpose (Kolena et al. 1997; Bar‐Ami et al. 2006; Demirel et al. 2018). In this study, pseudopregnancy was successfully induced in immature rats using this hormonal protocol. The rats were quarantined for 1 week and housed in polysulphone cages with aspen shavings for bedding, under controlled conditions of 21–24°C, 45%–55% humidity and a 12‐h light/12‐h dark cycle, at the Laboratory Animals Breeding and Experimental Research Unit of the Faculty of Pharmacy, Gazi University. During the experimental period, all animals were provided with a standard pellet diet and water ad libitum. The rats were maintained according to the guidelines outlined in the Guide for the Care and Use of Laboratory Animals.

### 2.4 Induction of Pseudopregnancy Model

**FIGURE 1:**
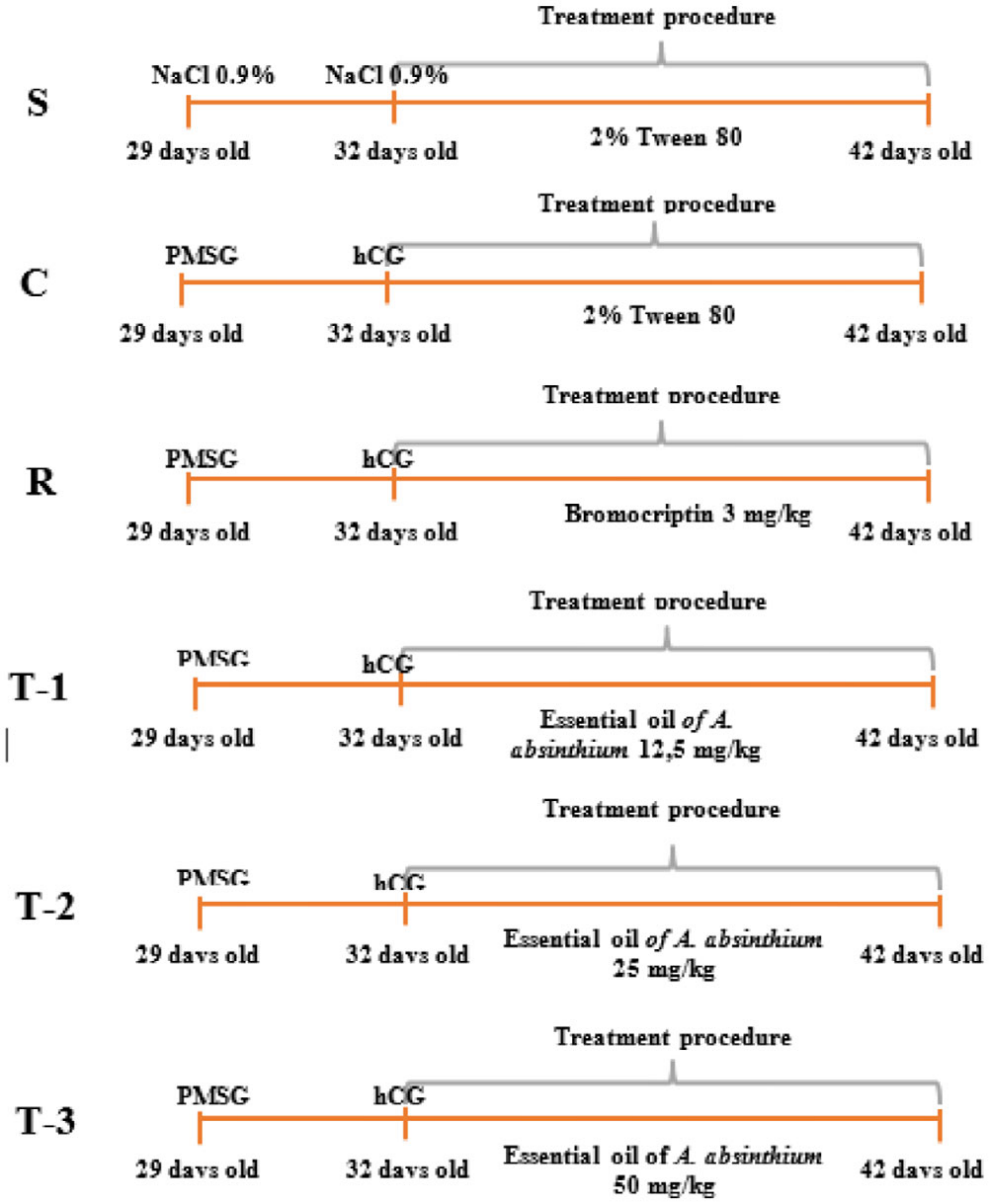
Flow chart of the experimental procedure.

A flowchart illustrating the experimental procedure is provided in Figure 1. The rats were randomly assigned to six groups, with each group consisting of six rats, as follows: sham (S) group, control (C) group, reference (R) group and three distinct treatment groups [T‐1 (12.5 mg/kg), T‐2 (25 mg/kg) and T‐3 (50 mg/kg)] treated with *A. absinthium* essential oil. Rats in the sham group received a subcutaneous injection of 0.9% NaCl. To establish the pseudopregnancy model, the 29‐day‐old rats were administered a single subcutaneous injection of 50 IU PMSG (Chronogest, Intervet, Türkiye). Three days following the PMSG injection, the 32‐day‐old rats were given a single subcutaneous dose of 20 IU hCG (Choragon, Erkim, Türkiye) (Bar‐Ami et al. 2006). The day of the hCG injection was designated as Day 0 of pseudopregnancy.

The oestrous stage of the rats was monitored throughout the experimental procedure. Vaginal smear samples were collected from all rats on Days 0, 5 and 10 following hCG administration. The stages of the oestrous cycle were classified as proestrus (characterized by oval and nucleated epithelial cells), oestrus (characterized by irregularly shaped cornified epithelial cells), metestrus (characterized by fragmented cornified epithelial cells and smaller, darker stained leukocytes) and dioestrus (characterized by nucleated epithelial cells with a predominance of leukocytes) (Cora et al. 2015).

### 2.5 The Treatment Procedure

The test materials were administered immediately following the hCG injection and continued for a duration of 10 days. The essential oil of *A. absinthium* was prepared in a 2% Tween 80 aqueous solution (Abdollahi et al. 2003; Abidi et al. 2018). A 2% Tween 80 aqueous solution (2 mL per os) was given to the sham and control groups. Bromocriptine (Parlodel, Meda Pharma, Türkiye) was administered to the reference group at a dose of 3 mg/kg per os. The treatment groups received doses of 12.5, 25 and 50 mg/kg of *A. absinthium* essential oil in the 2% Tween 80 aqueous solution, respectively.

### 2.6 Termination of the Experimental Procedure

On the 10th day of the treatment protocol, all rats were euthanized by exsanguination under general anaesthesia (10 mg/kg, i.p. xylazine hydrochloride and 80 mg/kg, i.p. ketamine hydrochloride). Uterine volume was estimated by measuring the length, width and height with a micrometre and applying the formula for the volume of an ellipsoid (π/6 × length × width × height). The uterine and ovarian tissues were excised and weighed using a precision scale. Additionally, the mammary chains were bilaterally dissected and weighed.

### 2.7 The Histopathological Evaluation of Mammary, Uterine and Ovarian Tissues

The mammary, uterine and ovarian tissues were immediately placed in a 10% neutral formaldehyde solution for 48 h. A pathologist grossed the samples to obtain appropriate tissue slices, which were then rinsed with 0.9% saline solution. The tissue samples were placed in cassettes and processed using an automated tissue processor. Subsequently, the samples were embedded in paraffin and sectioned into 5 µm‐thick slices. The slides were stained with haematoxylin and eosin (H&E) and examined blindly under a light microscope. Mammary alveolar developments, as well as uterine and ovarian tissues, were evaluated microscopically. The endometrial glands were counted, and the thickness of the myometrium was measured (five slides for each animal with five sections per slide) (Stewart et al. 2011). Additionally, the stage of the oestrous cycle was determined on the basis of the histological appearance of the uterine tissue (Dixon et al. 2014). The numbers of CL, regressed CL and tertiary follicles were counted in the ovarian tissues to assess ovarian follicular activity. Their numbers were counted at 7‐section intervals (whole ovaries were sectioned, resulting in at least 50 sections per ovary), and the number of various developmental follicles was multiplied by 7 to get an assessment of the total number of follicles per ovary (Gaytán et al. 2014). Mammary alveolar development was scored under ×10 magnification as follows: −: no alveoli; +: 5–10 alveoli; ++: 10–50 alveoli and +++: more than 50 alveoli (Demirel et al. 2018).

### 2.8 Statistical Analysis

Statistical analyses were performed using GraphPad Prism 6.0. The data are shown as the mean ± standard error of the mean (SEM). The one‐way analysis of variance (ANOVA) test followed by the Tukey multiple comparison test was used to determine the significance of differences between groups. The results were considered statistically significant at *p* < 0.05.

## 3 Results

### 3.1 Chemical Composition of the Essential Oil

GC–MS analysis identified a total of 60 compounds in the tested essential oil, as listed in Table 1. The identified compounds accounted for 90.5% of the total composition. The major constituent was *cis*‐chrysanthenyl acetate (17.8%), followed by sabinyl acetate (11.6%), terpinen‐4‐ol (6.2%), caryophyllene oxide (5.5%) and (*E*)‐nuciferol (5.5%).

**TABLE 1:** Chemical constituents of essential oil from *Artemisia absinthium* L. (%).

Compounds	RRI^a^	(%)
Sabinene	1124	0.1
Myrcene	1165	0.1
α‐Phellandrene	1168	tr^b^
α‐Terpinene	1183	tr
1,8‐Cineole	1211	0.1
2‐Hexanol	1225	tr
γ‐Terpinene	1249	0.2
*p*‐Cymene	1276	2.7
Terpinolene	1286	0.1
Perillen	1426	tr
*trans*‐Linalool oxide (furanoid)	1451	0.8
β‐Thujone	1454	0.1
*trans*‐Sabinene hydrate	1469	0.1
(*Z*)‐β‐ocimene oxide	1473	3.7
*cis*‐Linalool oxide (furanoid)	1479	0.6
Camphor	1535	0.1
Linalool	1548	4.5
*cis*‐Sabinene hydrate	1554	0.1
*trans‐p‐*Menth‐2‐ene‐1‐ol	1570	0.5
*cis*‐Chrysanthenyl acetate	1581	17.8
Bornyl acetate	1593	0.2
β‐Elemene	1601	0.6
Terpinen‐4‐ol	1612	6.2
*cis*‐*p*‐Menth‐2‐ene‐1‐ol	1634	0.3
α‐Thujenal	1645	0.2
Sabina ketone	1655	0.6
Sabinyl acetate	1663	11.6
Lavandulol	1681	0.5
*trans*‐Verbenol	1690	0.4
Carvotan acetone	1700	0.1
α‐Terpineol	1706	1.2
*trans*‐Sabinol	1711	0.5
β‐Selinene	1743	0.4
Phellandral	1745	0.2
Naphthalene	1769	1.4
Neryl isobutyrate	1783	1.4
*p*‐Methyl acetophenone	1800	1.0
Cumin aldehyde	1807	2.0
Perilla aldehyde	1810	0.4
Geraniol	1852	0.1
*p*‐Cymen‐8‐ol	1861	1.7
Neryl isovalerate	1866	1.8
Geranyl isovalerate	1886	1.4
*cis*‐Jasmone	1968	0.3
*(E)*‐12‐Norcaryophyll‐5‐ene‐2‐one	1996	0.6
Caryophyllene oxide	2017	5.5
*(E)*‐Nerolidol	2051	0.3
Humulene epoxide II	2074	0.5
Heneicosane	2100	0.1
Cumin alcohol	2121	0.8
Hexahydrofarnesyl acetone	2134	0.7
Spathulenol	2147	3.1
Nonanoic acid	2168	0.5
Thymol	2196	0.9
Selin‐11‐en‐4α‐ol	2263	4.6
Decanoic acid	2274	0.3
*(E)*‐Nuciferol	2355	5.5
Chamazulene	2436	0.2
Tetradecanoic acid	2698	0.3
*n*‐Hexadecanoic acid	2912	0.6
**Total identified**		**90.5**

^a^ Relative retention indices calculated against *n*‐alkanes.

^b^ Trace (<0.1%).

### 3.2 Vaginal Cytology

**FIGURE 2:**
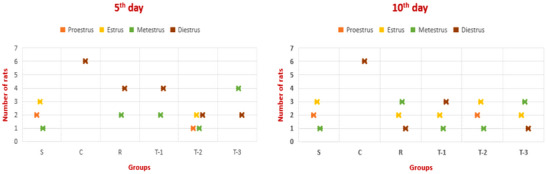
The oestrous cycle stages of the animals in each group on the 5th and 10th days of treatment. S: sham group; C: control group; R: reference group; T‐1: treatment group‐1 (12.5 mg/kg); T‐2: treatment group‐2 (25 mg/kg); T‐3: treatment group‐3 (50 mg/kg).

The cytological evaluation was conducted according to the classification described in Section 2. Vaginal smear samples obtained before the induction of the pseudopregnancy model indicated that the mean duration of the oestrous cycle was 4–5 days in all rats. This regular cycle was maintained throughout the experimental period in the S group. However, in the other groups, all animals except the T‐2 group were found to be in the luteal (metestrus–dioestrus) phase of the oestrous cycle following hormone administration on the fifth day of the treatment protocol. One animal in the T‐2 group was determined to be in proestrus, and two animals were in oestrous on the fifth day of treatment. On the 10th day of treatment, although the oestrous cycle was ongoing in the treatment groups, it was noted that all animals in the C group were in dioestrus (Figure 2).

**FIGURE 3:**
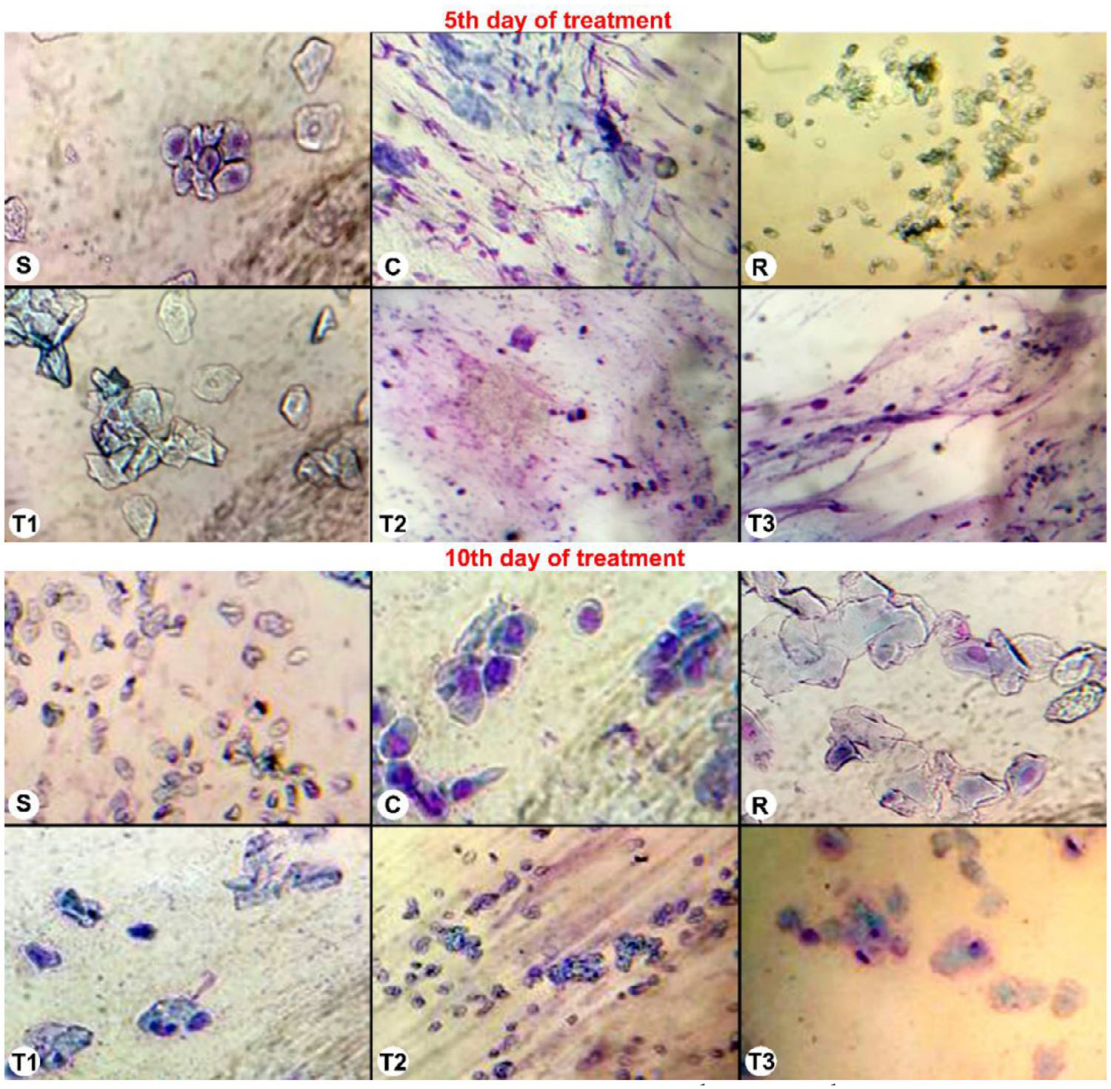
Vaginal cytology findings on the 5th and 10th of treatment. S: sham group; C: control group; R: reference group; T‐1: treatment group‐1 (12.5 mg/kg); T‐2: treatment group‐2 (25 mg/kg); T‐3: treatment group‐3 (50 mg/kg).

In the C group, nucleated epithelial cells characteristic of the luteal phase continued to be observed throughout the experimental period. In the treatment groups, irregularly shaped cornified squamous epithelial cells began to appear at the end of the procedure. Vaginal cytology during the oestrous stage is characterized by a predominance of superficial cells, particularly irregularly shaped cornified squamous epithelial cells. Thus, it was demonstrated that the oestrous cycle had resumed in these rats. These cellular alterations in the vaginal mucosal layer were similar across all treatment groups (Figure 3).

### 3.3 Mammary, Ovarian and Uterine Tissue Weights and Uterine Volume

**FIGURE 4:**
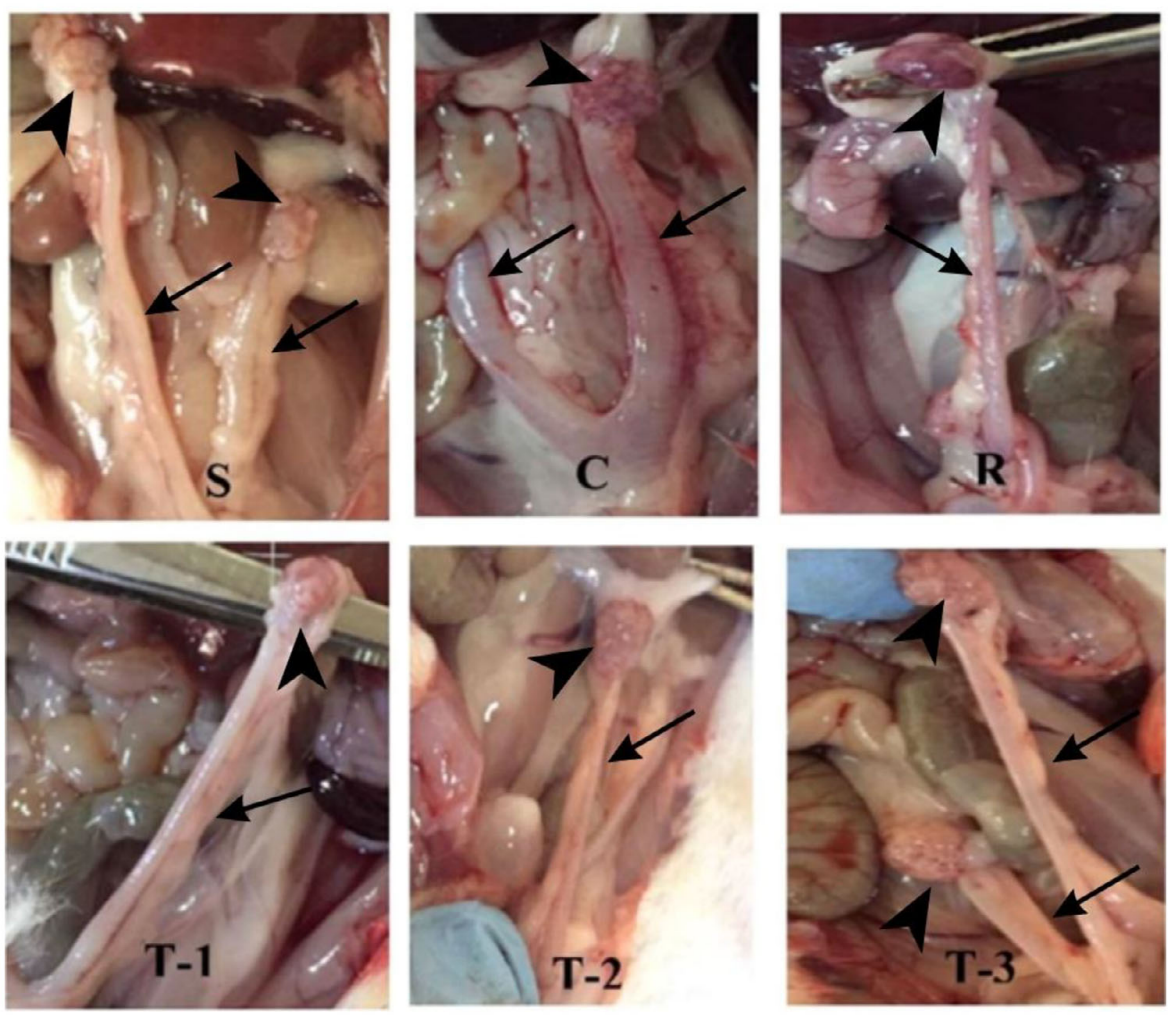
Macroscopic view of uterine (arrow) and ovarian (arrowhead) tissues. S: sham group; C: control group; R: reference group; T‐1: treatment group‐1 (12.5 mg/kg); T‐2: treatment group‐2 (25 mg/kg); T‐3: treatment group‐3 (50 mg/kg).

The macroscopic appearance of the uterine horns and ovaries is presented in Figure 4. No pathological alterations were observed in the uterine horns and ovaries of the S group. On the contrary, both uterine horns were enlarged in the C group compared to the other groups. Treatment with *A. artemisia* essential oil resulted in better macroscopic recovery compared to the C group. Although the mean uterine volume in the C group (267.90 ± 7.19 mm^3^) was higher than those in the treatment groups (R: 257.40 ± 6.31 mm^3^; T‐1: 251.20 ± 12.82 mm^3^; T‐2: 251.20 ± 0.00 mm^3^ and T‐3: 259.10 ± 7.85 mm^3^), the difference was not statistically significant (Table 2).

**TABLE 2:** The weight of mammary gland, ovarian and uterine tissue and uterine volume.

	Mean ± SEM			
Groups	Mammary gland weight (g)	Ovarian weight (g)	Uterine weight (g)	Uterine volume (mm^3^)
**S**	1.30 ± 0.09	0.07 ± 0.00**	0.22 ± 0.02	251.2 ± 3.97
**C**	1.70 ± 0.12	0.22 ± 0.04	0.25 ± 0.01	267.90 ± 7.19
**R**	1.48 ± 0.20	0.13 ± 0.02	0.22 ± 0.02	257.40 ± 6.31
**T‐1**	1.37 ± 0.02	0.16 ± 0.02	0.17 ± 0.00	251.20 ± 12.82
**T‐2**	1.29 ± 0.07	0.12 ± 0.01*	0.16 ± 0.02	251.20 ± 0.00
**T‐3**	1.48 ± 0.15	0.13 ± 0.01	0.17 ± 0.01	259.10 ± 7.85

*Note*: The experimental groups were compared with the control group (*p < 0.05; **p < 0.01); SEM: standard error of the mean; S: sham group; C: control group; R: reference group; T‐1: treatment group‐1 (12.5 mg/kg); T‐2: treatment group‐2 (25 mg/kg); T‐3: treatment group‐3 (50 mg/kg).

Ovarian weights were significantly different in S (0.07 ± 0.00 g, *p* < 0.001) and T‐2 (0.12 ± 0.01 g, *p* < 0.05) groups when compared to the C group (0.22 ± 0.04 g). Among the treatment groups, the greatest reduction in ovarian weight was observed in the T‐2 group. Although uterine weights were lower in the treatment groups, the differences were not statistically significant (*p *> 0.05) (Table 2).

**FIGURE 5:**
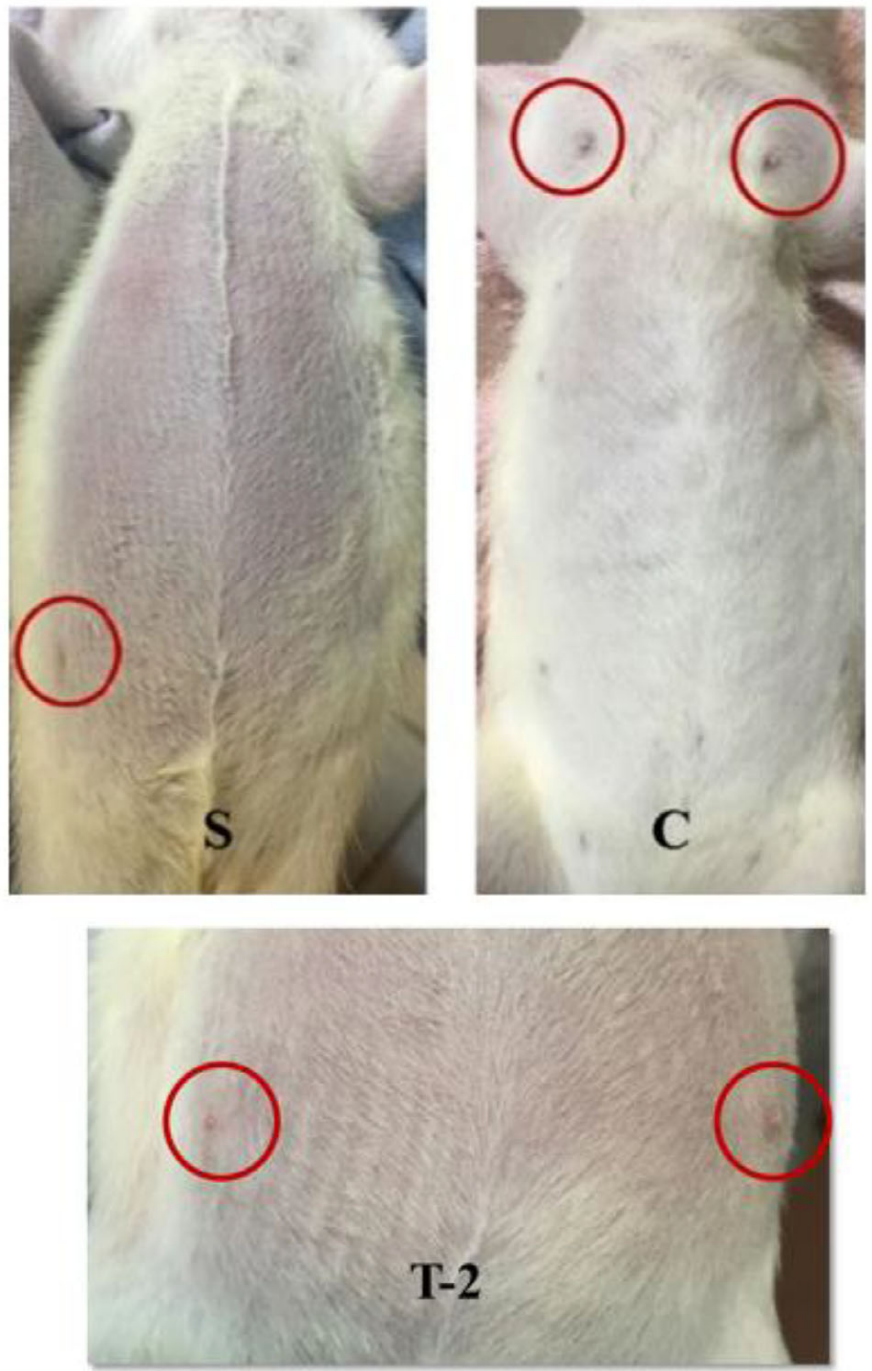
Macroscopic view of mammary glands. S: sham group; C: control group; T‐2: treatment group‐2 (25 mg/kg).

Due to pseudopregnancy induction, mammary gland enlargement was observed in the C group. However, mammary gland development in the treatment groups, particularly in the T‐2 group, was lower than in the C group (Figure 5). No statistically significant difference was found between the groups in terms of mammary gland weight (*p *> 0.05). Nevertheless, the highest values were recorded in the control group, whereas the lowest were observed in the T‐2 group (Table 2).

### 3.4 Histopathological Analysis

**FIGURE 6:**
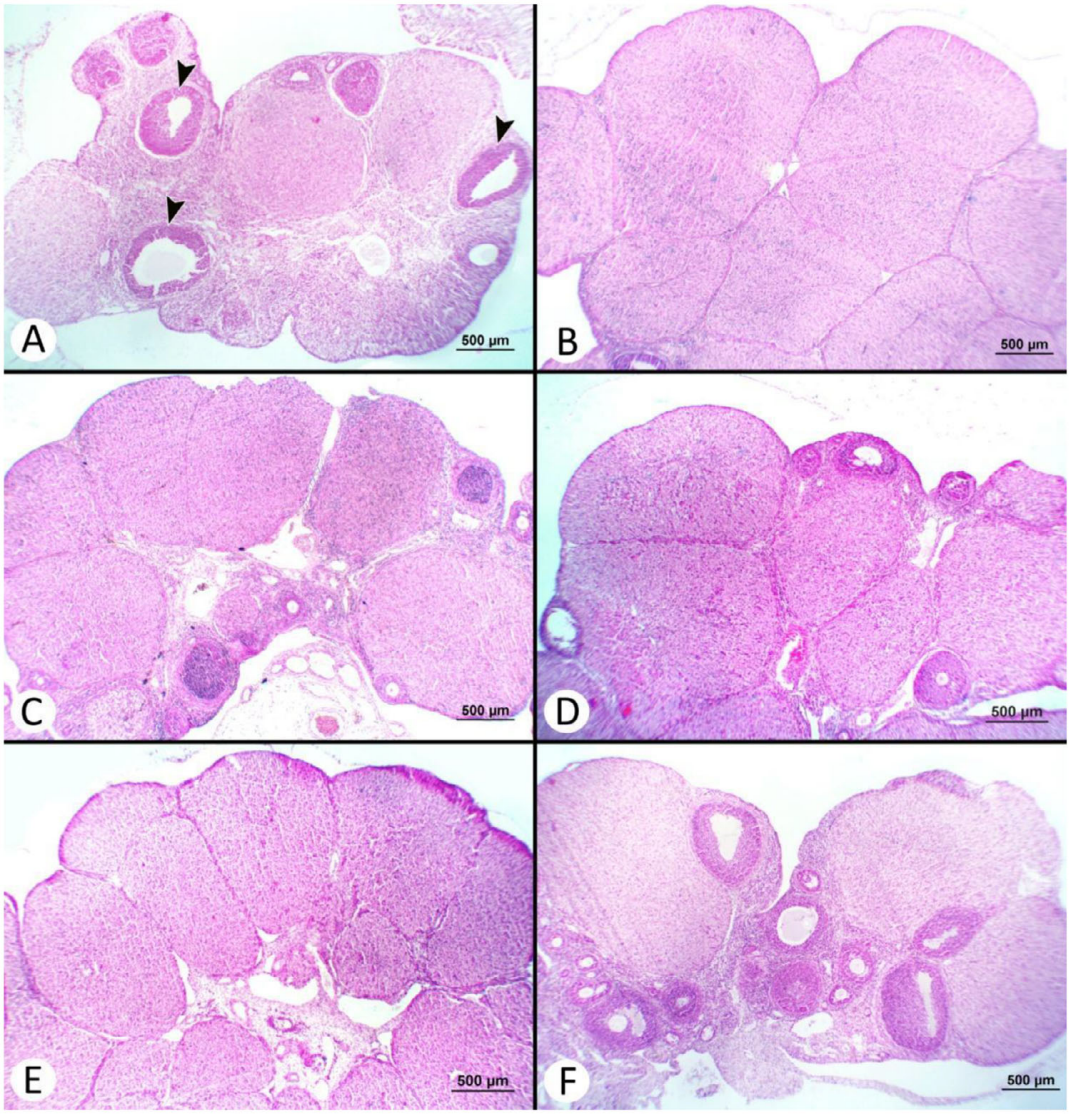
Histopathological view of ovarian tissues (H&E × 50): (A) The appearance of multiple tertiary follicle (arrowheads) developments in the sham group. (B) Microscopic view of the corpus luteum in the control group. (C) Microscopic view of a section of the ovary in the reference group. (D) Microscopic view of a section of the ovary in a treatment group of 12.5 mg/kg. (E) Multiple active and regressing corpora lutea in the treatment group of 25 mg/kg. (F) Follicular development in the treatment group of 50 mg/kg.

**FIGURE 7:**
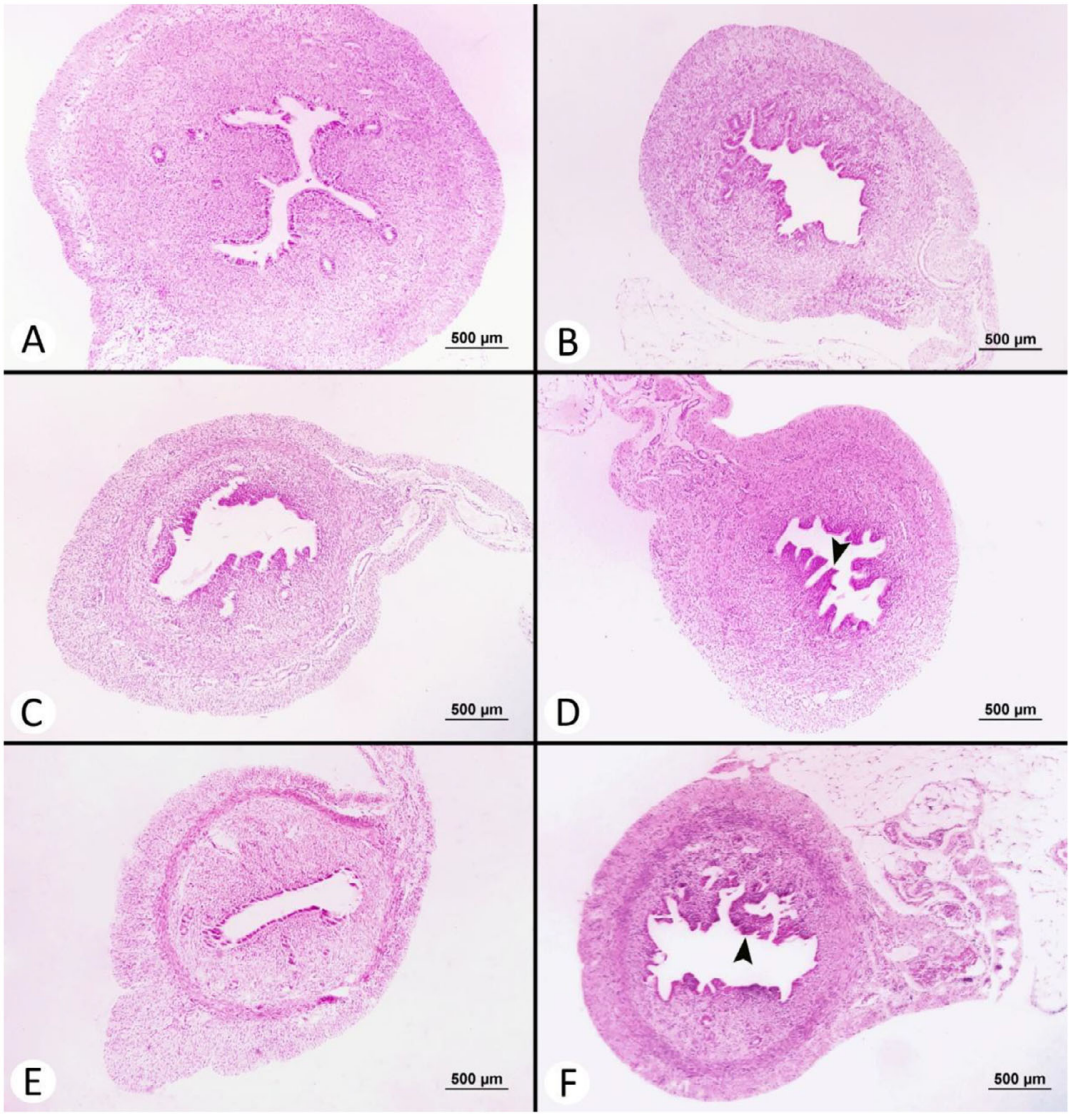
Microscopic view of uterine tissues (H&E × 50): (A) Apoptosis of the endometrial epithelium and development of the endometrial glands in the sham group. (B) Histological view of the uterus in the control group. (C) Desquamation of the endometrial epithelium in the reference group. (D) Mucosal plica formation toward the luminal space due to hyperplasia of the endometrial epithelium (arrowhead) in the treatment group of 12.5 mg/kg. (E) Endometrial epithelium lined with columnar epithelium in the treatment group of 25 mg/kg. (F) Appearance of mucosal plica formation (arrowhead) in the treatment group of 50 mg/kg.

**FIGURE 8:**
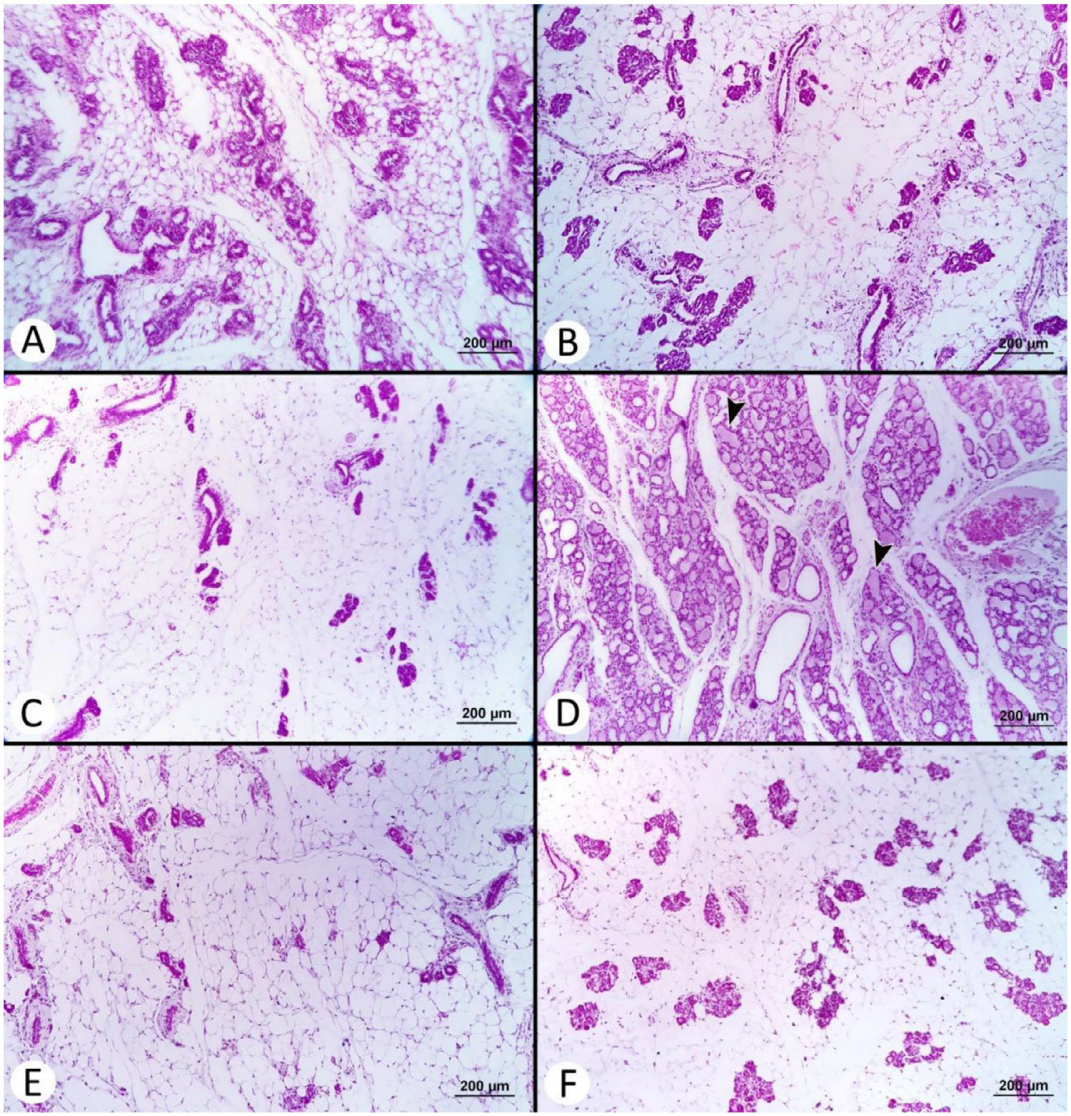
Microscopic view of mammary tissues (H&E × 50): (A) Appearance of prominent mammary alveoli in the sham group. (B) Microscopic view of the mammary gland in the control group. (C) Appearance of inactive mammary glands in the reference group. (D) The appearance of abundant milk synthesis within the active acini in the mammary gland (arrowheads) in the treatment group of 12.5 mg/kg. (E) Appearance of atrophic mammary glands in the treatment group of 25 mg/kg. (F) Microscopic view of the mammary gland in the treatment group of 50 mg/kg.

Microscopic findings of the ovarian, uterine and mammary tissues are summarized in Table 3. A significant difference was observed in the total CL count (*p* < 0.05). The total CL count was found to be the lowest in the S (4.67 ± 1.09) and T‐3 (9.33 ± 2.01) groups, and the highest in the C group (24.17 ± 6.27). No significant differences were found among the groups in terms of the number of regressed CL (*p* > 0.05); however, the highest number of regressed CL was observed in the reference group (R) (3.33 ± 1.12) and treatment group 1 (T‐1) (3.50 ± 0.76), with the lowest count in the S group (1.00 ± 0.26). Mucosal plica in the epithelium of the endometrium was notably present in the C and T‐1 groups. In these groups, the endometrial epithelium exhibited hyperplastic changes, and the endometrial glands were observed to be significantly enlarged. All rats in the S group were in the follicular phase, whereas the luteal phase was detected in all animals in the C group. Both follicular and luteal phases were observed in the treatment and reference groups. No significant difference was noted in the number of endometrial glands (*p* > 0.05). The least alveolar development was observed in the R and T‐2 groups. Histopathological images of the ovarian, uterine and mammary tissues are presented in Figures 6, 7 and 8, respectively.

**TABLE 3:** The histopathological parameters of ovarian and uterine tissues and mammary glands.

Parameters	S	C	R	T‐1	T‐2	T‐3
**Total corpus luteum**	32.69 ± 2.11^c^	170.01 ± 9.21^a^	108.02 ± 6.25^abc^	138.01 ± 5.65^ab^	105.12 ± 6.42^abc^	66.02 ± 4.02^bc^
**Regressed corpus luteum**	7.01 ± 1.53	20.01 ± 2.81	24.02 ± 2.12	24.58 ± 1.52	11.72 ± 2.10	12.91 ± 1.25
**Tertiary follicle**	35.10 ± 2.14	18.69 ± 3.72	35.00 ± 3.14	23.31 ± 2.82	18.69 ± 2.92	17.50 ± 1.77
**The number of endometrial glands**	7.67 ± 1.12	6.83 ± 1.01	8.00 ± 1.26	7.50 ± 0.76	8.83 ± 1.70	9.00 ± 1.29
**Thickness of myometrium**	238.33 ± 24.28	248.33 ± 19.56	255.00 ± 29.07	263.33 ± 24.59	265.00 ± 22.17	256.67 ± 17.26
**Mammary alveolar development**	2.00 ± 0.26	1.83 ± 0.31	1.50 ± 0.22	2.00 ± 0.26	1.50 ± 0.55	1.83 ± 0.31

*Note*: Means with different superscripts (^a–c^) within a row are different (*p* < 0.01); S: sham group; C: control group; R: reference group; T‐1: treatment group‐1 (12.5 mg/kg); T‐2: treatment group‐2 (25 mg/kg); T‐3: treatment group‐3 (50 mg/kg).

## 4 Discussion

Pseudopregnancy is not a disease but a syndrome characterized by pregnancy‐like symptoms. Although the condition often resolves spontaneously without the need for medical intervention, its effects on mammary tissue may persist, potentially leading to mastitis, mammary dermatitis or even breast cancer. Therefore, maintaining hormonal balance is essential in the treatment of pseudopregnancy. Various pharmacological agents have been utilized for its management, including anti‐prolactins, oestrogens, progestogens, serotonin agonists and dopamine agonists. The primary mechanism regulating prolactin secretion in pseudopregnancy involves the activation of dopaminergic neurons in the hypothalamus, which suppresses prolactin release. The use of ergot alkaloids (e.g., bromocriptine and cabergoline), which act as dopaminergic agonists on the hypothalamic–pituitary axis, underscores the role of prolactin in the etiopathogenesis of the syndrome. Progestins have been found to be ineffective in fully treating pseudopregnancy and are associated with a high recurrence rate. Moreover, their use is limited due to a wide range of serious side effects, making them unsuitable for pseudopregnancy treatment. In recent years, medical therapies have primarily focused on dopamine agonists and serotonin antagonists, with dopamine agonists being the preferred option due to their longer duration of action and minimal side effects. Notably, prolactin secretion is regulated by multiple neurotransmitters and hormones, further emphasizing the complexity of its modulation (Gobello et al. 2001b; Gobello 2006; Ramsey 2017; Root et al. 2018).

Previous studies have demonstrated that pseudopregnancy in experimental animals can be induced through various methods, including mating with vasectomized males, artificial mechanical stimulation of the vaginal–cervical region (Garrels et al. 2018; Wake et al. 2023) or administration of exogenous hormones in rats (Bar‐Ami et al. 2006; Eyster 2012; Demirel et al. 2018). Mechanical stimulation has been shown to trigger bi‐daily surges of prolactin, which subsequently promote ovarian progesterone secretion, leading to the establishment of pseudopregnancy without actual copulation (Poletini et al. 2012; Stump et al. 2014). This hormonally sustained dioestrus phase typically lasts for approximately 10–12 days (Stump et al. 2014). Alternatively, pseudopregnancy can be induced hormonally in immature rats, whose ovaries primarily consist of a relatively homogeneous population of follicles, predominantly antral follicles (40%–50%) and lack CLs. This model utilizes PMSG and hCG administration to initiate and regulate luteal activity (Kolena et al. 1997; Bar‐Ami et al. 2006; Demirel et al. 2018). In the current study, pseudopregnancy was established in immature rats via hormonal induction using PMSG and hCG.

The primary regulatory mechanism of prolactin secretion involves the activation of prolactin‐inhibiting dopaminergic neurons in the hypothalamus. Bromocriptine and cabergoline reduce prolactin secretion due to their strong dopamine D2‐receptor agonist activity. Similarly, serotonin antagonists indirectly inhibit prolactin secretion by stimulating endogenous dopamine release. Although dopamine agonists selectively suppress prolactin secretion, they are costly medications (Gobello et al. 2001a, 2006; Ramsey 2017; Root et al. 2018).

Given the high cost and adverse effects associated with current pharmacological treatments for pseudopregnancy, alternative medical approaches are being explored, with natural products emerging as promising candidates. Several medicinal plants, including *Pulsatilla alpina* subsp. *apiifolia* (Scop.) Nyman, *Ferula asafoetida* H.Karst., *Urtica dioica* L., *Thuja orientalis* L. and *M. chamomilla* L., were reported to be effective in pseudopregnancy (Özyurtlu and Alaçam 2005). Previous studies have demonstrated the dopaminergic effects of these plants (Martin et al. 1988; Park et al. 2014; Bharath Kumar et al. 2017; Bisht et al. 2017; Kabiri et al. 2019), suggesting that their beneficial effects on pseudopregnancy may be attributed to this mechanism. Similarly, the dopaminergic activity of *A. absinthium* L. has been reported in previous studies (Kharoubi et al. 2010; Zeraati et al. 2014). Building on this knowledge, our previous study investigated the therapeutic effects of different *A. absinthium* extracts on pseudopregnant rats (Demirel et al. 2018). Petroleum ether, dichloromethane and methanol extracts were administered orally at a dose of 100 mg/kg for 10 days. The results demonstrated that the petroleum ether extract exerted a modulatory effect on hormonal and histological changes associated with pseudopregnancy.

GC–MS analysis revealed that among the volatile compounds, *cis*‐chrysanthenyl acetate and β‐thujone were the major constituents in the most bioactive petroleum ether extract (Demirel et al. 2018). Consistently, the phytochemical analysis of the present study identified *cis*‐chrysanthenyl acetate as the predominant compound, accounting for 17.8% of the total composition. Additionally, sabinyl acetate, terpinen‐4‐ol, caryophyllene oxide and (*E*)‐nuciferol were identified as other major constituents. These findings align with previous studies, which have reported the presence of *cis*‐epoxyocimene, *cis*‐chrysanthenyl acetate, bornyl acetate, eucalyptol, myrcene, linalool, neryl butanoate, sabinene, *trans*‐sabinyl acetate, *trans*‐thujone, as well as α‐ and β‐thujone in the essential oil of *A. absinthium* (Pino et al. 1997; Orav et al. 2006; Rezaeinodehi and Khangholi 2008; Judzentiene and Budiene 2010; Bailen et al. 2013; Dhen et al. 2014; Julio et al. 2015; Batiha et al. 2020; Jiang et al. 2021).

In agreement with our findings, *cis*‐chrysanthenyl acetate has been reported as the major constituent in the essential oil of *A. absinthium*. Sharopov et al. (2012) analysed three *A. absinthium* samples from two different locations and found that *cis*‐chrysanthenyl acetate (17.9%) was the main component in one of the samples. Similarly, Bailen et al. (2013) reported a high concentration of *cis*‐chrysanthenyl acetate in the essential oil of *A. absinthium* grown under various environmental conditions. Comparable results were also documented by Judzentiene and Budiene (2010), Julio et al. (2015) and Obistioiu et al. (2014). However, other studies have identified chamazulene, bornyl acetate, neryl butanoate and sabinene as the dominant compounds in the essential oil of *A. absinthium* from different geographical regions (Pino et al. 1997; Sharopov 2012; Msaada et al. 2015). These variations in chemical composition can be attributed to differences in climatic conditions (rainfall, temperature and sunlight), geographical factors (altitude and soil composition) and the timing of harvest.

Thujone, a psychoactive compound, is known to induce hallucinations and enhance dopaminergic activity. Medicinal plants containing thujone have been traditionally used as abortifacients, contraceptives and uterotonics, as well as for the treatment of amenorrhoea and uterine carcinomas (Dhiman et al. 2012). However, prolonged use of *A. absinthium* essential oil may be toxic, leading to neurological disorders such as insomnia, convulsions and hallucinations. Judzentiene et al. (2012) investigated the toxicity of *A. absinthium* essential oil in relation to its chemical composition. Their findings indicated that the most toxic essential oils were those with high concentrations of *trans*‐sabinyl acetate and thujone, whereas samples containing an equivalent amount of sabinyl acetate but lacking thujone exhibited significantly lower toxicity. The European Food Safety Authority (EFSA) has classified α‐ and β‐thujone as potentially hazardous compounds in *A. absinthium*. However, previous studies have suggested that α‐ and β‐thujone concentrations ranging from 0% to 70.6% do not cause adverse effects (Szopa et al. 2020). In the present study, the β‐thujone content of the essential oil was determined to be 0.1%.

The findings of this study indicate that *A. absinthium* essential oil, administered at a dose of 25 mg/kg, exerted beneficial effects in the pseudopregnancy model in rats, leading to the return of cyclicity. This was evidenced by a reduction in uterine volume as well as decreases in the weights of the uterine, ovarian and mammary tissues compared to the control group, effects that are consistent with the follicular phase of the cycle. In addition, *A. absinthium* essential oil, indirectly via modulation of the oestrous cycle, induced histological alterations in the ovarian and mammary tissues in the treatment groups.

The normal oestrous cycle in rats lasts between 4 and 5 days and consists of four distinct stages: proestrus (12 h), oestrous (12 h), metestrus (21 h) and dioestrus (57 h) (Paccola et al. 2013; Cora et al. 2015). In pseudopregnancy, following ovulation, the dioestrus phase is prolonged by 13–18 days, during which the CLs become persistent and continue to secrete progesterone (Abd‐Elkareem 2017; Anderson and Musah 2013). During the initial days of pseudopregnancy, the progesterone‐dominant phase resembles that of gestation, with the CL being regulated by prolactin secretion. The nocturnal surge of prolactin plays a key role in stimulating the neuroendocrine pathway in pseudopregnant rats (Terkel 1988). These alterations in the oestrous cycle can be assessed through vaginal cytology (Cora et al. 2015). Pseudopregnancy is characterized by an irregular oestrous cycle, in which the nucleated epithelial cells of the dioestrus phase persist for an extended period (Stump et al. 2014; Demirel et al. 2018).

In the present study, vaginal cytology was evaluated on Days 0, 5 and 10 of the treatment protocol in pseudopregnant rats. Throughout the experimental period, nucleated epithelial cells characteristic of the dioestrus phase were consistently observed in all rats of the C group. In the treatment groups, however, irregularly shaped cornified squamous epithelial cells were detected at the end of the experiment, suggesting the initiation of the oestrous cycle. These cytological findings were similar across all treatment groups. Consequently, it was concluded that vaginal cytology alone was not a decisive factor in determining the effects of *A. absinthium* essential oil on pseudopregnancy.

Although embryo implantation does not occur in cases of pseudopregnancy, histomorphological changes are observed in the uterine tissue (Abd‐Elkareem 2017). These changes include nonspecific trauma‐related decidual cell differentiation, as well as hyperaemia and oedema in the uterine tissue, particularly evident during the mid‐stage of pseudopregnancy (Peel et al. 1979; Anderson and Musah 2013). The elevation of plasma progesterone levels results in mucosal plica formation, glandular development, epithelial proliferation and thickening of the uterine wall. Characteristic findings of the late stage of pseudopregnancy include epithelial proliferation, crypt formation, increased mucosal plica and glandular hypertrophy (Albers et al. 2015; Abd‐Elkareem 2017).

In this study, the number of CLs was significantly higher in the C group compared to the treatment groups. Consistent with previous studies, increased mucosal plica and glandular hypertrophy in the endometrium were notably observed in both the C and treatment group one (T‐1). Mucosal plica was also present in some cases within the other treatment groups. The previous research has indicated that mammary epithelial cell proliferation occurs during the metestrus stage, as seen in pseudopregnant females (Hvid et al. 2012). In the current study, mammary gland, alveolar and ductal developments were significantly enhanced in the C and T‐1 groups, whereas mammary tissue recovery was noted in the T‐2 and R groups. Overall, a dose of 25 mg/kg of *A. absinthium* essential oil was determined to be effective on uterine and mammary tissue in pseudopregnant rats.

A limitation of this study is the potential effect of natural puberty maturation, as the animals started the experiment in pre‐puberty. Therefore, some of the observed changes may reflect not only the effects of the experimental intervention but also normal developmental hormonal changes associated with puberty. Future clinical studies are needed to clarify these interactions and confirm the applicability of the findings to reproductive physiology.

## 5 Conclusion

The 25 mg/kg dose of *A. absinthium* essential oil was found to be effective in improving the pseudopregnancy model induced in rats. The outcome of the phytochemical analysis has shown that *A. absinthium* essential oil, which is rich in *cis*‐chrysanthenyl acetate, sabinyl acetate, terpinen‐4‐ol, caryophyllene oxide and (*E*)‐nuciferol, displayed therapeutic activity in terms of improving mammary development. Future studies are recommended to focus on elucidating the mechanisms at the hormonal (progesterone and prolactin), cellular and molecular levels to help further validate and expand the potential applications of *A. absinthium* essential oil in veterinary medicine.

## Author Contributions

**Mürşide Ayşe Demirel**: conceptualization, methodology, formal analysis, writing – original draft, writing – review and editing, software. **İpek Süntar**: conceptualization, methodology, formal analysis, writing – original draft, writing – review and editing, software. **Gökhan Zengin**: methodology, investigation. **Ali Osman Çeribaşı**: methodology, investigation. **Kevser Taban**: writing – original draft.

## Ethics Statement

The study was approved by the Experimental Animal Ethics Committee of Gazi University (G.U.ET‐17.015).

## Conflicts of Interest

The authors declare no conflicts of interest.

## Peer Review

The peer review history for this article is available at https://publons.com/publon/10.1002/vms3.70582.

## Data Availability

The data supporting the findings of this study are available from the corresponding author upon reasonable request.
